# EXAMINING AND COMPARING THE CLINICAL CHARACTERISTICS OF ADULTS WITH PERSISTING POST-CONCUSSION SYMPTOMS PRESENTING FOR OUTPATIENT REHABILITATION FOLLOWING A MILD TRAUMATIC BRAIN INJURY OR A MINIMAL HEAD INJURY

**DOI:** 10.2340/jrm.v57.43506

**Published:** 2025-07-01

**Authors:** Linda FORDAL, Grant L. IVERSON, Julia E. MAIETTA, Alexander OLSEN, Cathrine EINARSEN, Simen B. SAKSVIK, Toril SKANDSEN

**Affiliations:** 1 Department of Neuromedicine and Movement Science, Norwegian University of Science and Technology (NTNU), Trondheim, Norway; 2 Clinic of Rehabilitation, St. Olav’s University Hospital, Trondheim, Norway; 3 Department of Physical Medicine and Rehabilitation, Harvard Medical School, Boston, MA, USA; 4 Department of Physical Medicine and Rehabilitation, Spaulding Rehabilitation Hospital and the Schoen Adams Research Institute at Spaulding Rehabilitation, Charlestown, MA, USA; 5 Home Base, A Red Sox Foundation and Massachusetts General Hospital Program, Charlestown, MA, USA; 6 Mass General for Children Sports Concussion Program, Waltham, MA, USA; 7 Department of Psychology, Norwegian University of Science and Technology (NTNU), Trondheim, Norway; 8 Department of Research and Development, St. Olav’s University Hospital, Trondheim, Norway

**Keywords:** brain concussion, post-concussion syndrome, secondary care centres, rehabilitation, physical and rehabilitation medicine, persisting post-concussion symptoms, minimal head injury

## Abstract

**Objective:**

First, to describe a clinical sample with persisting post-concussion symptoms after a mild injury to the head. Second, to explore whether patients who sustained a mild traumatic brain injury differed from those with a minimal head injury (no loss of consciousness, no post-traumatic amnesia, no neuroimaging findings).

**Design:**

Cross-sectional clinic-referred sample.

**Subjects:**

178 adult patients with persisting post-concussion symptoms referred to outpatient rehabilitation.

**Methods:**

Main outcome measures were Rivermead Post-Concussion Symptoms Questionnaire, Glasgow Outcome Scale-Extended, and Return-to-work status.

**Results:**

In the total sample, previous health problems, daily headaches, fatigue, and depressive symptoms were frequent. Most had functional disability on the Glasgow Outcome Scale-Extended and had not returned to full-time work. The mean Rivermead Post-Concussion Symptoms Questionnaire total score was 29. Only 5 patients had intracranial traumatic injuries. Some 45% had sustained a minimal head injury. Patients with minimal head injury and mild traumatic brain injury had different causes of injury and acute care but were comparable regarding symptom burden and functional limitations.

**Conclusion:**

Clinicians treating persisting post-concussion symptoms may need to target physiological, psychological, and social factors. Many had an injury too mild to meet criteria for a traumatic brain injury, but the clinical phenotype was similar, supporting further research on the mildest head injuries.

## LAY ABSTRACT

First, we described the symptoms and functioning of 178 adults who were referred for rehabilitation due to persisting symptoms after a mild head injury. Second, almost half of the patients had suffered an injury too mild to meet criteria for a traumatic brain injury; they had a minimal head injury. We compared these 2 patient groups. Results showed that health problems before the injury were reported by both groups. Headaches, fatigue, and mental health problems after the injury were also common in both groups. Most were not working full-time and had many symptoms interfering with their daily lives. The patients who had suffered a minimal head injury thus had a similar symptom burden and level of functioning after the injury as those with a mild traumatic brain injury. Abnormal findings on computed tomography or magnetic resonance imaging were uncommon. Healthcare workers treating persisting symptoms after mild head injuries need to target physiological, psychological, and social factors.

Clinicians working with rehabilitation of patients who have persisting post-concussion symptoms (PPCS) and functional limitations following a presumed mild traumatic brain injury (MTBI) know that they represent a heterogeneous patient group. Many of these patients never presented to an emergency department in the acute phase, and were typically referred long after experiencing a head injury (1). This group of patients needs evidence-based healthcare, but their clinical profiles remain poorly understood (2). More than a decade ago, Luoto and colleagues illustrated the problem of selection bias in MTBI research. They concluded: “Studying carefully selected samples is often necessary to address specific research questions, but such studies have serious limitations in terms of translating research findings into clinical practice” (3). There is a vast literature regarding patients with head injuries who have been brought to the emergency department (ED) or have been admitted to the hospital; however, there is a paucity of studies from outpatient clinics (2).

Another clinical observation is that it may be unclear whether a patient with PPCS after a head injury actually sustained a traumatic brain injury (TBI), with signs of acute brain dysfunction or other evidence of brain pathology present in the acute stage (4). These injuries, which do not meet TBI criteria, have typically not been given a name. However, some terms that may include them are “minor head injury”, “mild head injury”, “concussion” and, according to newly published diagnostic criteria for MTBI, “suspected MTBI” (5). On the Head Injury Severity Scale (HISS), which is widely used in Scandinavia, such head injuries are categorized as a “minimal head injury” (MHI) (6, 7), which is the term hereafter used in this paper. These patients have typically not been enrolled in research studies and thus are under-represented and understudied – yet clinically important and in need of care (8). When referring to all patients, i.e. those with both minimal head injury/MHI and mild traumatic brain injury/MTBI, the overarching term “mild head injury” is used.

The first aim of this study was to describe the demographics, clinical characteristics, symptoms reporting, and functional limitations of patients referred to specialized healthcare due to PPCS after a mild head injury (MTBI or MHI). The second aim was to compare the patients *not* meeting established criteria for MTBI (i.e., patients with MHI), with those who did meet diagnostic criteria for MTBI, in an effort to increase knowledge and better understand the healthcare needs of patients with MHI.

## Methods

### Participants

We included patients, aged 16 to 65 years, who were referred to the Clinic of Rehabilitation at St. Olav's University Hospital, Trondheim, Norway, for evaluation and treatment for PPCS. Participants were mainly referred from general practitioners. Referrals also came from hospital physicians, mostly neurologists. The patients were enrolled between April 2019 and April 2024. To be included, patients had to have sustained a mild head injury within the past 5 years. Patients were not included in this study if they (*i*) had severe communication problems; (*ii*) their symptoms were considered better explained by another condition; or (*iii*) they had severe somatic, neurological, psychiatric, or substance abuse disorders deemed likely to complicate follow-up and outcome assessments, as assessed by the physician at the first consultation. The injury was retrospectively categorized as MTBI or MHI at the first consultation by the physician, based on information given by the patient. TBI was defined as an alteration in brain function, operationalized as loss of consciousness, amnesia for the event, slow or confused behaviour, or other evidence of brain pathology (e.g., traumatic lesions on neuroimaging), caused by an external force (4) and was further defined as mild by the WHO Collaborating Centre Task Force criteria for MTBI (9). These criteria specifically include: (*i*) Glasgow Coma Scale (GCS) score of 13–15 at presentation; (*ii*) loss of consciousness (LOC) < 30 min; and (*iii*) post-traumatic amnesia < 24 h. The term MTBI was chosen, comprising injuries with and without traumatic lesions on neuroimaging. Head injuries that did not meet full criteria for MTBI were categorized as MHI. Thus, the individuals in the MHI group experienced an injury to the head with no self-reported acute alteration or loss of consciousness, no amnesia for the event, and no neuroimaging findings. This definition follows the HISS (6) definition of a minimal head injury (also used in the Scandinavian Guidelines for initial management of head injuries [7]) characterized by: (*i*) GCS score of 15; (*ii*) no LOC; and (*iii*) no amnesia for the event. In our sample, the GCS score was in many cases not recorded, which we interpreted as equal to 15 given the absence of symptoms or other information to suggest a lower GCS score. All participants (MTBI and MHI) experienced post-concussion symptoms within the first week after their head injury and PPCS were defined as endorsing at least 1 moderate or 3 mild symptoms on the Rivermead Post-Concussion Symptoms Questionnaire (RPQ) (10) at the first consultation. The term PPCS was used for all participants admitted and included in the study, even though not all (77%) had experienced their symptoms for longer than 3 months – which is a common lower limit for PPCS. Our use of the term PPCS includes PPCS-like symptoms, as it is unclear whether those with MHI have sustained an MTBI or not. The study was approved by the regional committee for research ethics (REK 2018/2159).

### Study procedures

Patients were informed about the study on their first visit to the Rehabilitation Clinic. During this visit, a baseline semi-structured interview was conducted by the treating physician. This interview covered demographics, pre-injury health and functioning, information concerning the injury, and symptoms and functioning at the time of the first consultation. Informed consent was collected separately, after the first visit. If patients accepted, they, or the legal guardians of those younger than 18 years of age, signed an electronic consent form. After consenting, they received additional digital questionnaires regarding their health and functioning.

### Measures

*Demographics and pre-injury functioning.* Education, marital status, parental status, employment status, and a history of learning difficulties were all self-reported by the participant. Learning difficulties referred to reading or writing difficulties or receiving special education. Previous head injuries and symptoms thereafter, as well as pre-injury mental health problems and sleep problems, were also self-reported. No diagnostic criteria or confirmation by medical records were applied or required for the above-mentioned problems. When a previous head injury was reported, the physician asked detailed questions about loss of consciousness, the presence of amnesia, level of acute and post-acute care required, and any necessary neuroimaging to evaluate whether MTBI or MHI was most likely.

*Injury-related characteristics and symptoms in the acute phase.* Place and mechanism of injury, other injuries, early symptoms, signs, and method of contact with healthcare were self-reported. CT and MRI findings were based on clinical imaging. When performed, CT had most often been obtained at St. Olav’s University Hospital in the acute stage, and MRI usually at a later stage, in most cases with a 1.5 Tesla system. Neuroimaging findings were categorized into “no findings” when self-reported as normal. When reported as positive, findings were ascertained by the physician at the Rehabilitation Clinic, through a review of the radiological report. The findings reported are intracranial traumatic injuries.

*Symptoms and functioning at the first Rehabilitation Clinic consultation.* PPCS was measured using the RPQ and the RPQ total score was an outcome measure. The RPQ is a self-report questionnaire measuring the severity of the following 16 symptoms during the past 24 h, compared with before the injury: headaches, dizziness, nausea/vomiting, noise sensitivity, sleep disturbance, fatigue, being irritable, feeling depressed, feeling frustrated, forgetfulness, poor concentration, taking longer to think, blurred vision, light sensitivity, double vision, and restlessness. The respondent rates each of these symptoms based on the following options: (0) Not at all; (1) No more of a problem than before the injury; (2) A mild problem; (3) A moderate problem; or (4) A severe problem. The total score ranges from 0 to 52, where higher scores represent a higher severity of symptoms (10). Because we were only interested in problems that were in excess of baseline symptomatology, we transformed 1-point answers to 0 points.

Global functioning was our second outcome measure, assessed by the physician with the Glasgow Outcome Scale-Extended (GOS-E) (11, 12), using the structured interview. The GOS-E questions include: level of consciousness, self-efficacy in and out of the home, work, social activities, relationships, and return to a normal life. GOS-E scores range from 1–8. Our sample included GOS-E scores from 5 to 8 where scores of 5 and 6 were classified as lower and upper moderate disability (low score) and GOS-E scores of 7 and 8 were classified as lower and upper good recovery (high score).

The third outcome measure was negative change in work status, categorized as “yes” in participants who worked full-time before the injury, but who were not working full-time at the first consultation at the Rehabilitation Clinic (hereafter termed negative Return-to-work status). Full-time employment referred to working or studying 80% or more of a full-time job (37.5 h a week; i.e., a minimum of 30 h per week). Work status was self-reported by the participant.

### Questionnaires

Six questionnaires were administered electronically. The *Epworth Sleepiness Scale* measures daytime sleepiness, more specifically the likelihood of dozing off or falling asleep in different everyday situations, on a scale from 0 (would never doze/fall asleep) to 4 (high chance of dozing/falling asleep) (13). A score of 11 or higher represents excessive daytime sleepiness (14). A recent review of its reliability considers it an important tool in sleep assessment and states that the test–retest reliability is similar or better than in other tools measuring excessive daytime sleepiness (15) The *Fatigue Severity Scale* consists of 9 statements measuring feelings of fatigue on a scale from 1 (completely disagree) to 7 (completely agree) (16). The maximum score is 63. Each total score is divided by the number of items. We chose to use 5 points as the cut-off for excessive fatigue, because the previous cut-off of 4 has been considered over-inclusive (17). The scale was validated in a large Swiss cohort and was judged to have both excellent test–retest reliability and internal consistency (18) The *Insomnia Severity Index* measures degree of insomnia with 7 questions. The maximum score is 28, and each question is rated from 0 to 4. A score of 15 or above represents moderate to severe insomnia, and the instrument has shown good reliability and validity as well as excellent internal consistency (19). The *Generalized Anxiety Disorder-7 Scale* assesses problems with anxiety over the past week. The maximum score is 21 and each statement is answered using a scale from 0 (not at all) to 3 (almost every day). A score of 10 points or higher represents moderate to severe anxiety over the past week. The instrument is much used and has been shown to be both reliable and valid (20). The *Patient Health Questionnaire* assesses depression with 9 statements that are answered from 0 (not at all) to 3 (almost every day). The maximum score is 27 points. A score of 10 points is the cut-off for moderate to severe depression. The questionnaire is considered sensitive and specific in measuring the severity of depressive symptoms and to be a reliable and a valid tool in assessing depression (21). The *Post-Traumatic Stress Disorder Checklist* measures symptoms of post-traumatic stress in the past week with a list of 21 problems rated on a scale from 0 (not at all) to 4 (very much), where 84 is the maximum score (22). A total score of 32 was used as cut-off for clinically significant post-traumatic stress. A recent systematic review showed acceptable internal consistency and test–retest reliability (23).

### Statistical analyses

χ^2^ tests were used to compare the MTBI group and the MHI group on categorical variables. Post-hoc analyses were also performed to compare men and women. Independent *t*-tests were used for continuous variables with a normal distribution, while Mann–-Whitney *U* tests were used for continuous variables without a normal distribution. Shapiro–Wilk tests, histograms, and skewness and kurtosis tests were used to assess normality. Standard deviations were presented when data were normally distributed and interquartile ranges when they were not. *P-*values not corrected for multiple comparisons are reported in the tables and in the text. Alpha was set at 0.05 for determination of statistical significance. Further, *p*-values still significant after Bonferroni correction for multiple comparisons are marked in the tables with an asterisk. Analyses were performed in IBM SPSS Statistics version 28.0.1.0 and version 30.0.0.0 (IBM Corp, Armonk, NY, USA), and version 17 of Stata (StataCorp LLC, College Station, TX, USA). Missing data were handled using a pairwise deletion/complete case approach.

## Results

### Demographic characteristics

A total of 202 patients were invited to this study, and 24 of the invited patients were not included or later excluded due to: no registered written consent (*n* = 20), or withdrawal from the study (*n* = 4). Consequently, 178 patients were included in the current study, 98 in the MTBI group (55%), and 80 in the MHI group (45%). Their median age at injury was 37 years. Most of the sample were women (70%), had higher education (59%), had a domestic partner (72%), and were fully employed before their injury (90%). There were no significant differences between the groups on demographic characteristics (Table I).

**Table I:** Demographic information and pre-injury health and functioning

Item	Total sample (*n* = 178)	MTBI (*n* = 98)	MHI (*n* = 80)	*p*-value
Age at injury, Md (IQR)	36.5 (25.0–49.0)	42.5 (24.8–53.0)	34.0 (26.0–42.0)	0.072
Sex, female, *n* (%)	124 (69.7)	71 (72.4)	53 (66.3)	0.371
University/College degree, *n* (%)	103 (58.9)	51 (52.6)	52 (66.7)	0.060
Marital status, partner^1^, *n* (%)	126 (71.6)	71 (72.4)	55 (70.5)	0.777
Caregiver for children, *n* (%)	62 (35.0)	36 (36.7)	26 (32.9)	0.596
Fully employed^2^, *n* (%)	160 (89.9)	85 (86.7)	75 (93.8)	0.123
Learning difficulties^3^, *n* (%)	23 (15.3)	14 (17.1)	9 (13.2)	0.516
Previous MTBI or MHI, *n* (%)	91 (51.7)	46 (47.4)	45 (57.0)	0.208
Symptoms > 2 weeks	27 (16.1)	8 (8.6)	19 (25.3)	**0.004**
Symptoms at the time of current injury	2 (1.2)	1 (1.1)	1 (1.3)	–^5^
Headache problem, *n* (%)	81 (46.3)	41 (42.3)	40 (51.3)	0.235
At the time of injury	39 (22.3)	17 (17.7)	22 (27.8)	0.109
Family history of headache, *n* (%)	56 (37.6)	30 (38.0)	26 (37.1)	0.917
Mental health problem, *n* (%)	73 (42.2)	39 (41.1)	34 (43.6)	0.737
At the time of injury	23 (13.9)	13 (14.6)	10 (13.2)	0.789
Sleep problem, *n* (%)	59 (35.8)	32 (35.2)	27 (36.5)	0.860
At the time of injury	41 (25.0)	22 (24.2)	19 (26.0)	0.786
Problems at time of injury, total, *n* (%)^4^	72 (40.4)	36 (36.7)	36 (45.0)	–

Md: median; IQR: interquartile range; MTBI: mild traumatic brain injury; MHI: minimal head injury. The number of people with missing, unknown, or cannot answer values for each variable are as follows: education *n* = 3; civil status *n* = 2; caregiver for children *n* = 1; school difficulties *n* = 28; head injuries *n* = 2; headache problem *n* = 3; family history of headache *n* = 29; mental health problem *n* = 5; sleep problem *n* = 13

1 Partner = boyfriend/girlfriend, married, or cohabitation.

2 Fully employed = works/studies 80% or more of a full-time position, i.e., a minimum of 30 h per week.

3 School difficulties = reading, writing, or learning difficulties or special education.

4 Problems at time of injury, total = 1 or more of the following problems at the time of the current injury: symptoms from a previous head injury, headache problem, mental health problem, sleep problem.

5 Too few participants to calculate a *p*-value.

* Bonferroni correction for multiple comparisons, *p* = 0.003. *P*-values significant after Bonferroni correction are marked with an asterisk. Statistically significant *p*-values are marked with bold font.

### Pre-injury self-reported health and functioning

In the total sample, pre-injury health problems such as previous head injuries (52%), headaches, (46%) and mental health problems (42%) were frequently reported, and there were no significant differences in -proportions reporting these problems between the 2 groups (see Table I). The MHI group more frequently reported having experienced symptoms longer than 2 weeks after a previous head injury (MHI group = 25%, MTBI group = 9%, *p* = 0.003). For the majority of those with pre-injury problems, the problems were not ongoing at the time of injury. Some 40% of the entire sample confirmed at least 1 of the above-mentioned pre-injury problems at the time of their current injury (MHI group = 45%, MTBI group = 37%; see Table I).

**Table II:** Injury-related characteristics and symptoms in the acute phase after injury

Item	Total sample (*n* = 178)	MTBI (*n* = 98)	MHI (*n* = 80)	*p*-value
Location of injury, *n* (%)				0.002^*^
Sport/recreation	45 (26.5)	19 (20.4)	26 (33.8)	
Home	40 (23.5)	26 (28.0)	14 (18.2)	
Street/road	39 (22.9)	30 (32.3)	9 (11.7)	
Job/school	23 (13.5)	8 (8.6)	15 (19.5)	
Public place	23 (13.5)	10 (10.8)	13 (16.9)	
Mechanism of injury, *n* (%)				< 0.001^*^
Fall	89 (50.0)	56 (57.1)	33 (41.3)	
Hit object	50 (28.1)	15 (15.3)	35 (43.8)	
Violence	9 (5.1)	5 (5.1)	4 (5.0)	
Traffic	30 (16.9)	22 (22.4)	8 (10.0)	
Post-traumatic amnesia, *n* (%)	86 (49.1)	86 (88.7)	0 (0)	**–**
Loss of consciousness, *n* (%)	28 (19.2)	28 (39.4)	0 (0)	**–**
Other injuries, *n* (%)	31 (18.8)	19 (21.1)	12 (16.0)	0.403
Symptom onset immediately, *n* (%)	128 (74.0)	78 (80.4)	50 (65.8)	**0.030**
Symptom onset, *n* (%)				0.086
Immediately	128 (74.0)	78 (80.4)	50 (65.8)	
< 24 h	23 (13.3)	9 (9.3)	14 (18.4)	
> 24 h	22 (12.7)	10 (10.3)	12 (15.8)	
Neck pain in the acute phase, *n* (%)	55 (44.4)	34 (52.3)	21 (35.6)	0.061
Vomiting in the acute phase, *n* (%)	17 (10.7)	16 (19.0)	1 (1.3)	< 0.001^*^
Influence of alcohol^1^, *n* (%)	28 (16.1)	21 (22.1)	7 (8.9)	**0.018**
Influence of other drugs^1^, *n* (%)	0 (0)	0 (0)	0 (0)	**–**
Days to 1st contact physician^2^, Md (IQR)	0.0 (0.0–3.0)	0.0 (0.0–2.0)	1.0 (0.0 – 5.0)	< 0.001^*^
Highest level of acute treatment *n* (%)				< 0.001^*^
General practitioner	75 (42.6)	28 (28.9)	47 (59.5)	
Municipal emergency clinic	56 (31.8)	32 (33.0)	24 (30.4)	
Hospital, not admitted	13 (7.4)	8 (8.2)	5 (6.3)	
Hospital, observation < 24 h	14 (8.0)	12 (12.4)	2 (2.5)	
Hospital, admitted > 24 h	18 (10.2)	17 (17.5)	1 (1.3)	
CT, *n* (%)	103 (58.2)	69 (70.4)	34 (43.0)	< 0.001^*^
Findings^3^ (of all participants)	5 (2.8)	5 (5.1)	0 (0)	–
Findings^3^ (of those who had CT)	5 (4.9)	5 (7.2)	0 (0)	**–**
Days to CT^4^, Md (IQR)	2.0 (0.0–7.3)	0 (0.0–7.0)	4.5 (1.0–11.0)	**0.011**
MRI, *n* (%)	108 (61.7)	60 (62.5)	48 (60.8)	0.814
Findings^3^ (of all participants)	2 (1.1)	2 (2.0)	0 (0)	–
Findings^3^ (of those who had MRI)	2 (1.9)	2 (3.3)	0 (0)	–
Weeks to MRI^5^, Md (IQR)	17 (7.0–33.0)	18.5 (9.0–32.8)	15.0 (5.0–34.0)	0.223

MTBI: mild traumatic brain injury; MHI: minimal head injury; CT: computed tomography; MRI: magnetic resonance imaging; Md: median; IQR: interquartile range. The number of people with missing, unknown, or cannot answer values for each variable are as follows: place of injury *n* = 8; post-traumatic amnesia *n* = 3; loss of consciousness *n* = 32; other injuries *n* = 13; symptom onset *n* = 5; acute neck pain *n* = 54; acute vomiting *n* = 19; influence of alcohol *n* = 4; influence of other drugs *n* = 14; days to first contact physician *n* = 7; highest treatment level acute phase *n* = 2; CT *n* = 1; MRI *n* = 3.

1 Self-reported influence of alcohol or other drugs.

2 Days from injury to first contact with physician (includes by telephone).

3 Imaging findings presented are intracranial traumatic injuries.

4 Number of days from injury to CT.

5 Number of weeks from injury to MRI.

* Bonferroni correction for multiple comparisons, *p* = 0.004. *P*-values significant after Bonferroni correction are marked with an asterisk. Statistically significant *p*-values are marked with bold font.

### Injury-related characteristics

There were several differences between groups on injury-related characteristics and symptom reporting. The location (*p =* 0.002) and mechanism (*p =* < 0.001) of injury differed between the groups (Table II). The MHI group more often sustained their injury in a sports or recreational setting (MHI group: 34%, MTBI group: 20%), were more often hit by an object (44% vs 15%), were less often involved in traffic accidents (10% vs 22%) and falls (41% vs 57%), and were also less likely to report that they were under the influence of alcohol at the time of injury (9% vs 22%, *p =* 0.018). In contrast, the MTBI group more often had an immediate onset of symptoms after the injury (80% vs 66%, *p =* 0.030), more often sought healthcare on the day of the injury (68% vs 33%, *p <* 0.001) and were more likely to be put under observation or admitted to the hospital (30% vs 4%, *p <* 0.001) than the MHI group – who typically saw only their general practitioner in the acute phase (60% vs 29%, *p <* 0.001).

In the MTBI group, more had been referred for head CT (70% vs 43%, *p <* 0.001), and they were referred for CT sooner than in the MHI group (median 0 vs 5 days, *p* = 0.011). Only 5 patients in the MTBI group (3% of all participants) had intracranial findings on CT. MRI was performed in 108 patients (62%), at a median of 17 weeks after the injury. Two patients (1% of all participants) had intracranial traumatic lesions on MRI, and in 1 of them, CT had already shown the intracranial lesion. All patients with findings on neuroimaging met the clinical criteria for MTBI in the acute stage, except 1 where a chronic subdural hematoma evolved over time (see Table II).

### Symptoms and functioning on the first visit to the Rehabilitation Clinic

The median time from injury to the first visit to the Rehabilitation Clinic was 6 months in the total sample (MTBI group = 5.8 months, MHI group = 6.4 months). Headache complaints in the past 3 months were reported by 91% of all participants and the median number of headache days per month was the maximum of 30 (Table III). Dizziness and neck pain were common symptoms, as 68% of the participants confirmed current dizziness and 46% reported neck pain. The mean RPQ total score was 30 (SD 12) for the MTBI group and 28 (SD 10) for the MHI group. Most participants had GOS-E scores of 5 or 6 (MTBI group = 76%, MHI group = 86%). There was a difference in Return-to-work status between the groups such that the MHI group had not returned to working full-time after their injury to the same degree as the MTBI group, i.e., they significantly more often had a negative return-to-work status (MHI group = 73%, MTBI group = 56%, *p =* 0.026; Table III).

**Table III:** Symptoms and functioning on the first consultation at the Rrehabilitation Clinic

Item	Total sample (*n* = 178)	MTBI (*n* = 98)	MHI (*n* = 80)	*p*-value
Time to 1st consultation, months, Md (IQR)	5.9 (3.5–10.6)	5.8 (3.4–10.6)	6.4 (3.7–10.7)	0.809
RPQ total, M (SD)	29.4 (11.1)	30.4 (12.1)	28.2 (9.9)	0.203
GOS-E, Md (IQR)	6.0 (5.0–6.0)	6.0 (5.0–6.25)	6.0 (5.0–6.0)	0.297
GOS-E, *n* (%)				0.073
Low: disability (5–6)	143 (80.3)	74 (75.5)	69 (86.3)	
High: good recovery (7–8)	35 (19.7)	24 (24.5)	11 (13.8)	
Overall symptoms, *n* (%)				0.691
Better	93 (55.7)	51 (58.0)	42 (53.2)	
Worse	14 (8.4)	6 (6.8)	8 (10.1)	
No change	60 (35.9)	31 (35.2)	29 (36.7)	
Headaches past 3 months, *n* (%)	160 (91.4)	88 (90.7)	72 (92.3)	0.709
Headache severity past 3 months, *n* (%)				0.602
Mild	61 (39.1)	31 (37.3)	30 (41.1)	
Moderate	83 (53.2)	44 (53.0)	39 (53.4)	
Severe	12 (7.7)	8 (9.6)	4 (5.5)	
Headache duration past 3 months, *n* (%)				0.565
< 4 h	24 (18.0)	11 (15.1)	13 (21.7)	
4 h–1 day	82 (61.7)	45 (61.6)	37 (61.7)	
1–3 days	11 (8.3)	6 (8.2)	5 (8.3)	
> 3 days	16 (12.0)	11 (15.1)	5 (8.3)	
Headache days past month, Md (IQR)	30 (19.5–30.0)	30 (20.8–30.0)	30 (16.5–30.0)	0.747
Neck pain, *n* (%)	66 (45.5)	33 (42.9)	33 (48.5)	0.494
Dizziness, *n* (%)	97 (68.3)	53 (69.7)	44 (66.7)	0.695
Employment full time^1^, *n* (%)	48 (27.3)	31 (32.3)	17 (21.3)	0.101
Negative Return-to-work status^2^, *n* (%)	112 (63.6)	54 (56.3)	58 (72.5)	**0.026^3^**

MTBI: mild traumatic brain injury; MHI: minimal head injury; Md: median; IQR: interquartile range; M: mean; SD: standard deviation; RPQ: Rivermead Post-Concussion Symptoms Questionnaire; GOS-E: Glasgow Outcome Scale-Extended. The number of people with missing, unknown, or cannot answer values for each variable are as follows: RPQ *n* = 10; overall symptoms *n* = 11; headache *n* = 3; headache intensity *n* = 22; headache duration *n* = 45; headache days past month *n* = 49; neck pain *n* = 33; dizziness *n* = 36; employment full time *n* = 2. Statistically significant *p*-values are marked with bold font.

1 Employment full-time = employed/studies 80% or more of a full-time position, i.e., a minimum of 30 h per week.

2 Negative Return-to-work status = worked or studied full-time before the injury but not at the first rehabilitation clinic consultation.

3 Bonferroni correction for multiple analyses, *p* = 0.004. *P*-values significant after Bonferroni correction are marked with an asterisk.

### Questionnaires on health and functioning

In total, 60% of the participants completed questionnaires (*n* = 106). When we compared those who completed questionnaires with those who did not, no differences were found in terms of demographics, pre-injury, injury-related, or outcome measures. Among those with questionnaire data, 52% reported excessive fatigue and 42% reported moderate to severe depressive symptoms (Table IV). There were no significant differences between the MTBI group and the MHI group on any of the measures assessing clinically relevant problems. There was, however, a difference between groups on total score on the Epworth Sleepiness scale (median for the MTBI group = 8 points, the MHI group = 6 points, *p =* 0.006; Table IV).

**Table IV:** Questionnaires completed after the first consultation

Questionnaires and measures	Total sample *n* = 178	MTBI *n* = 98	MHI *n* = 80	*p*-value
Completion of ≥ 1 questionnaire, *n* (%)	106 (59.6)	56 (57.1)	51 (63.0)	0.469
Time to complete^1^, weeks, Md (IQR)	3.0 (1.0–10.0)	3.0 (1.0–11.5)	4.0 (1.0–9.5)	0.915
Epworth Sleepiness Scale, total score, Md (IQR)	7.0 (5.0–11.5)	8.0 (6.0–12.0)	6.0 (3.0–9.3)	**0.006^2^**
Excessive daytime sleepiness, ≥ 11 points, *n* (%)	30 (29.7)	20 (36.4)	10 (21.7)	0.109
Fatigue Severity Scale total score, Md (IQR)	5.0 (3.9–5.9)	4.9 (3.8–6.0)	5.1 (4.0–5.9)	0.817
Excessive fatigue ≥ 5 points, *n* (%)	55 (51.9)	28 (50.0)	27 (54.0)	0.681
Insomnia Severity Index, Md (IQR)	8.0 (4.0–14.0)	8.0 (5.0–13.0)	7.0 (3.0–14.0)	0.596
Moderate/severe insomnia ≥ 15 points, *n* (%)	16 (15.8)	8 (14.5)	8 (17.4)	0.696
Generalized Anxiety Disorder-7 Scale, Md (IQR)	5.0 (3.0–7.0)	5.0 (3.0–7.8)	5.0 (2.0–6.0)	0.308
Moderate/severe anxiety ≥ 10 points, *n* (%)	13 (12.3)	7 (12.5)	6 (12.0)	0.938
Patient Health Questionnaire (PHQ-9), Md (IQR)	8.0 (6.0–12.0)	8.5 (6.0–13.0)	7.0 (5.8–11.0)	0.180
Moderate/severe depression ≥ 10 points, *n* (%)	44 (41.5)	26 (46.4)	18 (36.0)	0.277
Posttraumatic Stress Disorder Checklist, Md (IQR)	12.0 (8.0–24.0)	15.0 (9.0–27.0)	11.5 (5.8–17.3)	0.075
Posttraumatic stress ≥ 32 points, *n* (%)	13 (12.9)	9 (16.4)	4 (8.7)	0.252

MTBI: mild traumatic brain injury; MHI: minimal head injury; Md: median; IQR: interquartile range. The number of people with missing, unknown, or cannot answer values for each variable are as follows: Epworth Sleepiness Scale *n* = 77; Fatigue Severity Scale *n* = 72; Insomnia Severity Index *n* = 77; Generalized Anxiety Disorder-7 Scale (GAD-7) *n* = 72; Patient Health Questionnaire (PHQ-9) *n* = 72, Posttraumatic Stress Disorder Checklist *n* = 77.

1 Time from first consultation to completion of questionnaires.

2 Bonferroni correction for multiple analyses, *p* = 0.004. Statistically significant *p*-values are marked with bold font. *P*-values still significant after Bonferroni correction are marked with an asterisk.

### Sex differences in symptoms and functioning before and after the injury

Among the study participants, 70% were women. Because of this discrepancy, we performed additional analyses based on sex and reported significant differences (Table V). Women had higher education, as 64% of women reported a university or college degree, compared with 47% of the men (*p =* 0.038). Women also reported more pre-injury headaches (52% vs 31%, *p =* 0.011) and pre-injury mental health problems (48% vs 28%, *p =* 0.011) than men did. Men reported more often having 1 or several previous mild head injuries (65% vs 46%, *p =* 0.021) compared with women (no moderate or severe head injuries were reported). There were no significant differences between men and women on injury-related factors, symptom reporting, or functioning at the first Rehabilitation Clinic consultation. However, there were differences in reported symptoms in questionnaires. First, 60% of women reported excessive fatigue, compared with 29% of men (*p =* 0.004). Second, 46% of men reported excessive daytime sleepiness on the Epworth Sleepiness Scale, compared with 24% of women (*p =* 0.033; Table V).

**Table V:** Sex differences in symptoms and functioning before and after the injury

Item	Total sample	Women	Men	*p*-value
Sex, *n* (%)	–	124 (69.7)	54 (30.3)	–
Age, Md (IQR)	36.5 (25.0–49.0)	36.0 (25.0–48.8)	38.0 (24.0–49.3)	0.934
University/college degree, *n* (%)	103 (58.9)	78 (63.9)	25 (47.2)	**0.038^1^**
Pre-injury headache problem, *n* (%)	81 (46.3)	65 (52.4)	16 (31.4)	**0.011**
Headache problem at time of injury, *n* (%)	39 (22.3)	33 (26.8)	6 (11.5)	**0.026**
Family history of headaches, *n* (%)	56 (37.6)	46 (44.2)	10 (22.2)	**0.011**
Pre-injury mental health problems, *n* (%)	73 (42.2)	59 (48.4)	14 (27.5)	**0.011**
Previous MTBI / MHI, *n* (%)	91 (51.7)	56 (45.9)	35 (64.8)	**0.021**
Symptoms > 2 weeks	27 (16.1)	13 (11.3)	14 (26.4)	**0.013**
Fatigue Severity Scale, total score, Md (IQR)	5.0 (3.9–5.9)	5.3 (4.4–6.0)	4.2 (3.7–5.6)	**0.047**
Excessive fatigue ≥ 5 points, *n* (%)	55 (51.9)	47 (60.3)	8 (28.6)	0.004^*^
Epworth, excessive sleepiness ≥ 11 points, *n* (%)	30 (29.7)	18 (24.0)	12 (46.2)	**0.033**
Insomnia Severity Index, total score, Md (IQR)	8.0 (4.0–14.0)	7.0 (4.0–13.0)	11.5 (6.0–15.3)	**0.010**

Md: median; IQR: interquartile range; MTBI: mild traumatic brain injury; MHI: minimal head injury. The number of people with missing, unknown, or cannot answer values for each variable are as follows: University/college degree *n* = 3; pre-injury headache problem *n* = 3; family history of headaches *n* = 29; pre-injury mental health problems *n* = 5; previous mild head injury *n* = 2; Fatigue Severity Scale *n* = 72; Epworth Sleepiness Scale *n* = 77; Insomnia Severity Index *n* = 77.

1 Only factors with significant differences are reported in the table above, with the exception of age. Epworth = Epworth Sleepiness Scale, excessive daytime sleepiness. Statistically significant *p*-values are marked with bold font.

* *P*-values still significant after Bonferroni correction are marked with an asterisk. Bonferroni correction for multiple comparisons, *p* = 0.005.

## DISCUSSION

We examined clinical outcomes in an important, yet under-studied, group in head injury research: rehabilitation outpatients with PPCS after a mild head injury, and in particular the subgroup with an injury deemed too mild to meet diagnostic criteria for MTBI: those with MHI. In the total sample, there were frequent reports of pre-injury health problems, a high symptom burden, chronic headaches, depression and fatigue, functional disability, and a low Return-to-work rate. Despite the many symptoms and low functioning, exceptionally few had intracranial traumatic injuries. The MHI group comprised almost half of all participants. The MTBI and MHI groups were clinically similar when presenting to the clinic, yet the MTBI group more often had traffic accidents as cause of injury, reported more immediate symptoms after the injury, sought healthcare earlier, and were more often hospitalized and referred for acute neuroimaging, all supporting that the MTBI group had sustained a more severe injury.

### Demographics

A clear majority of the patients were women. Although more men sustain MTBI (24), women are more likely to develop PPCS, and female sex has consistently been found to be a risk factor for PPCS (25, 26). Some explanatory factors may be social and psychological (27, 28) and others biological (28–30). We do not currently have sufficient knowledge and understanding of the specific gendered outcome after MTBI. Further research in this area is needed.

Of all patients in this study, 59% had a university or college degree, and significantly more were women. This is, however, not very different from the general Norwegian population. In the age group corresponding to the median age of our participants (35–39 years), more than 50% have a university or college degree, and also in the general population, more are women (31).

### Pre-injury health and functioning

More than half of the participants had sustained 1 or more mild head injuries before the current injury; this was not surprising as we also included MHI. We do not know how prevalent MHI is in the general population as this group is excluded from most head injury research. However, we do know that having repeated head injuries is a potential prognostic factor for PPCS (25). While the prevalence of previous head injuries did not differ between groups, participants in the MHI group had more often experienced prolonged symptoms after a previous mild head injury (although these symptoms had ceased by the time of their current injury). This finding supports the view that some people may have an underlying, individual vulnerability for developing PPCS after an MHI. It may therefore be important to inquire about not only previous head injuries, but also about previous PPCS.

Pre-injury mental health problems were reported by 42% of the participants. This is in line with previous research, often showing that a history of mental health problems is a risk factor for developing PPCS after MTBI (32). The current study adds to this knowledge by including the patients without a definite MTBI. Headaches before the injury were also common, reported by almost half of the participants. Interestingly, all of the above-mentioned pre-injury problems were usually not ongoing problems at the time of injury, suggesting that even a remote history of health problems could constitute a long-term vulnerability for PPCS.

### Injury-related characteristics

There were marked differences between the groups regarding injury-related characteristics indicating that those with MHI had suffered a less severe injury. There were differences in immediate symptoms and acute treatment between the groups (e.g., time to seek healthcare, level of acute care, and referrals for neuroimaging), beyond the criteria originally separating the 2 groups into different injury severities.

An important finding in this study was the low rate of intracranial traumatic injuries on neuroimaging. Only 1–3% of the entire sample had documented intracranial traumatic injuries on CT or MRI. This is remarkably less than in the much cited studies from TRACK-TBI and CENTER-TBI, conducted in the emergency departments of large trauma centres, where CT showed traumatic lesions in around 30–50% of the participants (33, 34), and where 27% of patients with a normal CT in a TRACK-TBI study had MRI findings (35). Neuroimaging findings have been associated with higher risk of worse outcome (34). Given our selection of patients with a high symptom burden, a greater number of neuroimaging findings could have been expected. In patients who were categorized as MHI based on clinical characteristics, only 1 had findings on later imaging (a chronic subdural haematoma). It is further interesting that the cause of injury differed between the MTBI and MHI groups. Only in the MHI group was it common to report having hit the head against an object, a mechanism that potentially is less forceful, compatible with a milder injury. The underlying pathophysiology of ongoing PPCS is not well characterized, and it is debatable to what degree PPCS represents an underlying structural brain injury or a functional disorder (36). We consider our results to support the hypothesis that PPCS is mostly not indicative of brain injury. Rather, PPCS may be understood as a constellation of symptoms that are precipitated by the sensory input or the neurometabolic cascade induced by a mild head injury, but at a later stage may represent a dysregulation of brain functioning that can occur in the absence of a brain injury, and that is not specific to TBI. More research on the pathophysiology of PPCS is needed.

### Symptoms and functioning at the first consultation

On the first consultation at the Rehabilitation Clinic, the total sample reported a number of symptoms and problems as well as functional limitations. Notably, headaches were reported among virtually all participants, and most had daily headaches. This overwhelming frequency of post-traumatic headache likely reflects some degree of referral bias, whereby patients with chronic headaches are likely to be referred to this Rehabilitation Clinic. Teams treating patients with PPCS need expertise in the treatment of headache.

On their first visit, most participants had not returned to full-time work. This was not surprising, because long-term sick leave often leads to referrals to specialist healthcare. One contributing factor to the overall low Return-to-work status could be the high occurrence of post-traumatic headaches in our sample, which has been associated with lower rates of Return-to-work (38, 39). Another contributing factor could be mental health problems, because a large number of the participants reported depression and fatigue after the injury. Support for this association can be found in other studies (40–42). Hence, a joint focus on occupational needs and psychological status and coping after the injury is important in the rehabilitation of patients with PPCS. A surprising finding was that the MHI group had returned to full-time work significantly less than the MTBI group, despite the groups otherwise reporting a similar symptom burden and functioning. We do not have a clear explanation for this finding, and it possibly represents a random finding. More research is needed to confirm this result.

Many people with mild head injuries who subsequently develop PPCS only see their general practitioner in the acute phase and beyond. Early collaboration with specialty healthcare may be beneficial for individuals at risk of PPCS. Further, it highlights the important role of general practitioners; their identification of patients at risk for PPCS, and their advice on post-injury care.

### Limitations

First, most of the data gathered on pre-injury and injury-related factors were retrospectively collected self-reported data, thus there is a risk of recall bias. However, the patients were interviewed at the first opportunity, most often by 6 months after the injury. Second, the questionnaire response rate was lower than desired, around 60%, but analyses comparing those who replied to the questionnaires with those who did not revealed no differences in demographics, pre-injury, injury-related, or outcome measures between those with missing questionnaire data and those with complete data. Third, because our sample was derived from the healthcare-seeking population of mild head injury patients with PPCS who were referred to a rehabilitation clinic, findings do not generalize to the average person suffering PPCS. Last, we could have chosen the American Congress of Rehabilitation Medicine’s new diagnostic criteria for MTBI (5). However, because our study sample comprised many patients who never sought emergency healthcare, we did not have sufficiently specific information to utilize the new category “suspected MTBI”. Rather, we chose the term MHI, which is an established term in Scandinavian healthcare.

### Conclusion

We studied a large sample of patients referred to a specialized rehabilitation clinic because they had persisting symptoms following a mild head injury. These patients had significant pre-injury health problems, a heavy burden of post-injury persisting symptoms and functional disability. Nevertheless, there were very few neuroimaging findings. Nearly half of the patients did not fulfil the diagnostic criteria for MTBI; they had an MHI. Although the MHI group had sustained a milder injury than the MTBI group, the groups were comparable regarding symptom burden and functional limitations. This lack of association between injury severity and negative clinical outcome challenges the view that PPCS represents the direct neurobiological effects of a lingering TBI, and it suggests that a biopsychosocial approach (where physiology, behaviour, cognition, emotions, and factors related to social, working, and family life are considered) is best suited to help us further investigate the underlying mechanisms of PPCS and develop evidence-based treatment.

## References

[1] Gordon KE. The silent minority: insights into who fails to present for medical care following a brain injury. Neuroepidemiology 2020; 54: 235–242. 10.1159/000503579. 31614354

[2] Zogg CK, Haring RS, Xu L, Canner JK, Ottesen TD, Salim A, et al. Patient presentations in outpatient settings: epidemiology of adult head trauma treated outside of hospital emergency departments. Epidemiology 2018; 29: 885–894. 10.1097/ede.0000000000000900. 30063541 PMC6167152

[3] Luoto TM, Tenovuo O, Kataja A, Brander A, Öhman J, -Iverson GL. Who gets recruited in mild traumatic brain injury research? J Neurotrauma 2013; 30: 11–16. 10.1089/neu.2012.2611. 22909262

[4] Menon DK, Schwab K, Wright DW, Maas AI. Position statement: Definition of traumatic brain injury. Arch Phys Med Rehabil 2010; 91: 1637–1640. 10.1016/j.apmr.2010.05.017. 21044706

[5] Silverberg ND, Iverson GL, Cogan A, Dams OCK, Delmonico R, Graf MJP, et al. The American Congress of Rehabilitation Medicine diagnostic criteria for mild traumatic brain injury. Arch Phys Med Rehabil 2023; 104: 1343–1355. 10.1016/j.apmr.2023.03.036. 37211140

[6] Stein SC, Spettell C. The Head Injury Severity Scale (HISS): a practical classification of closed-head injury. Brain Injury 1995; 9: 437–444. 7550215 10.3109/02699059509008203

[7] Undén J, Ingebrigtsen T, Romner B. Scandinavian guidelines for initial management of minimal, mild and moderate head injuries in adults: an evidence and consensus-based update. BMC Med 2013; 11. 10.1186/1741-7015-11-50. 23432764 PMC3621842

[8] Coffeng SM, Jacobs B, Kim LJ, Ter Maaten JC, van der Naalt J. Incomplete recovery in patients with minor head injury directly discharged home from the emergency department: a prospective cohort follow-up study. BMJ Open 2022; 12: e057308. 10.1136/bmjopen-2021-057308. PMC9244716 35768088

[9] Carroll LJ, Cassidy JD, Holm L, Kraus J, Coronado VG. Methodological issues and research recommendations for mild traumatic brain injury: the WHO Collaborating Centre Task Force on Mild Traumatic Brain Injury. J Rehabil Med 2004; 43: 113–125. 10.1080/16501960410023877. 15083875

[10] King NS, Crawford S, Wenden FJ, Moss NEG, Wade DT. The Rivermead Post Concussion Symptoms Questionnaire: a measure of symptoms commonly experienced after head injury and its reliability. J Neurology 1995; 242: 587–592. 10.1007/BF00868811. 8551320

[11] Jennett B, Bond M. Assessment of outcome after severe brain damage. Lancet 1975; 1: 480–484. 10.1016/s0140-6736(75)92830-5. 46957

[12] Wilson L, Boase K, Nelson LD, Temkin NR, Giacino JT, Markowitz AJ, et al. A manual for the Glasgow Outcome Scale – extended interview. J Neurotrauma 2021; 38: 2435–2446. 10.1089/neu.2020.7527. 33740873 PMC8390784

[13] Johns MW. A new method for measuring daytime sleepiness: the Epworth sleepiness scale. Sleep 1991; 14: 540–545. 10.1093/sleep/14.6.540. 1798888

[14] Johns M, Hocking B. Daytime sleepiness and sleep habits of Australian workers. Sleep 1997; 20: 844–849. 10.1093/sleep/20.10.844. 9415943

[15] Scharf MT. Reliability and efficacy of the Epworth sleepiness scale: is there still a place for it? Nat Sci Sleep 2022; 14: 2151–2156. 10.2147/nss.S340950. 36536636 PMC9759004

[16] Krupp LB, LaRocca NG, Muir-Nash J, Steinberg AD. The fatigue severity scale: application to patients with multiple sclerosis and systemic lupus erythematosus. Arch Neurol 1989; 46: 1121–1123. 10.1001/archneur.1989.00520460115022. 2803071

[17] Lerdal A, Wahl A, Rustøen T, Hanestad BR, Moum T. Fatigue in the general population: a translation and test of the psychometric properties of the Norwegian version of the fatigue severity scale. Scand J Public Health 2005; 33: 123–130. 10.1080/14034940410028406. 15823973

[18] Valko PO, Bassetti CL, Bloch KE, Held U, Baumann CR. Validation of the fatigue severity scale in a Swiss cohort. Sleep 2008; 31: 1601–1607. 10.1093/sleep/31.11.1601. 19014080 PMC2579971

[19] Morin CM, Belleville G, Bélanger L, Ivers H. The Insomnia Severity Index: psychometric indicators to detect insomnia cases and evaluate treatment response. Sleep 2011; 34: 601–608. 10.1093/sleep/34.5.601. 21532953 PMC3079939

[20] Spitzer RL, Kroenke K, Williams JB, Löwe B. A brief measure for assessing generalized anxiety disorder: the GAD-7. Arch Intern Med 2006; 166: 1092–1097. 10.1001/archinte.166.10.1092. 16717171

[21] Kroenke K, Spitzer RL, Williams JB. The PHQ-9: validity of a brief depression severity measure. J Gen Intern Med 2001; 16: 606–613. 10.1046/j.1525-1497.2001.016009606.x. 11556941 PMC1495268

[22] Blevins CA, Weathers FW, Davis MT, Witte TK, Domino JL. The posttraumatic stress disorder checklist for DSM-5 (PCL-5): development and initial psychometric evaluation. J Trauma Stress 2015; 28: 489–498. 10.1002/jts.22059. 26606250

[23] Forkus SR, Raudales AM, Rafiuddin HS, Weiss NH, Messman BA, Contractor AA. The Posttraumatic Stress Disorder (PTSD) Checklist for DSM-5: a systematic review of existing psychometric evidence. Clin Psychol (New York) 2023; 30: 110–121. 10.1037/cps0000111. 37378352 PMC10292741

[24] Brazinova A, Rehorcikova V, Taylor MS, Buckova V, Majdan M, Psota M, et al. Epidemiology of traumatic brain injury in Europe: a living systematic review. J Neurotrauma 2021; 38: 1411–1440. 10.1089/neu.2015.4126. 26537996 PMC8082737

[25] Déry J, Ouellet B, de Guise É, Bussières È-L, Lamontagne M-E. Prognostic factors for persistent symptoms in adults with mild traumatic brain injury: an overview of systematic reviews. Syst Rev 2023; 12: 127. 10.1186/s13643-023-02284-4. 37468999 PMC10357711

[26] King NS. A systematic review of age and gender factors in prolonged post-concussion symptoms after mild head injury. Brain Injury 2014; 28: 1639–1645. 10.3109/02699052.2014.954271. 25265040

[27] Starkey NJ, Duffy B, Jones K, Theadom A, Barker-Collo S, Feigin V. Sex differences in outcomes from mild traumatic brain injury eight years post-injury. PLoS One 2022; 17: e0269101. 10.1371/journal.pone.0269101. 35622845 PMC9140230

[28] Mikolić A, van Klaveren D, Groeniger JO, Wiegers EJA, Lingsma HF, Zeldovich M, et al. Differences between men and women in treatment and outcome after traumatic brain injury. J Neurotrauma 2021; 38: 235–251. 10.1089/neu.2020.7228. 32838645

[29] Mauvais-Jarvis F, Bairey Merz N, Barnes PJ, Brinton RD, Carrero JJ, DeMeo DL, et al. Sex and gender: modifiers of health, disease, and medicine. Lancet 2020; 396: 565–582. 10.1016/S0140-6736(20)31561-0. 32828189 PMC7440877

[30] Casale R, Atzeni F, Bazzichi L, Beretta G, Costantini E, Sacerdote P, et al. Pain in women: a perspective review on a relevant clinical issue that deserves prioritization. Pain Ther 2021; 10: 287–314. 10.1007/s40122-021-00244-1. 33723717 PMC8119594

[31] Statistisk Sentralbyrå. Befolkningens utdanningsnivå. 2024 [cited 2024 13Nov]. Available from: https://www.ssb.no/utdanning/utdanningsniva/statistikk/befolkningens-utdanningsniva.

[32] Cassidy JD, Cancelliere C, Carroll LJ, Côté P, Hincapié CA, Holm LW, et al. Systematic review of self-reported prognosis in adults after mild traumatic brain injury: results of the international collaboration on mild traumatic brain injury prognosis. Arch Phys Med Rehabil 2014; 95: 132–151. 10.1016/j.apmr.2013.08.299. 24581902

[33] Mikolić A, Steyerberg EW, Polinder S, Wilson L, Zeldovich M, von Steinbuechel N, et al. Prognostic models for global functional outcome and post-concussion symptoms following mild traumatic brain injury: a Collaborative European NeuroTrauma Effectiveness Research in Traumatic Brain Injury (CENTER-TBI) Study. J Neurotrauma 2023; 40: 1651–1670. 10.1089/neu.2022.0320. 37078144 PMC10458380

[34] Yuh EL, Jain S, Sun X, Pisică D, Harris MH, Taylor SR, et al. Pathological computed tomography features associated with adverse outcomes after mild traumatic brain injury: a TRACK-TBI study with external validation in CENTER-TBI. JAMA Neurology 2021; 78: 1137–1148. 10.1001/jamaneurol.2021.2120. 34279565 PMC8290344

[35] Yue JK, Yuh EL, Korley FK, Winkler EA, Sun X, Puffer RC, et al. Association between plasma GFAP concentrations and MRI abnormalities in patients with CT-negative traumatic brain injury in the TRACK-TBI cohort: a prospective multicentre study. Lancet Neurol 2019; 18: 953–961. 10.1016/s1474-4422(19)30282-0.31451409

[36] Clark CN, Edwards MJ, Ong BE, Goodliffe L, Ahmad H, Dilley MD, et al. Reframing postconcussional syndrome as an interface disorder of neurology, psychiatry and psychology. Brain 2022; 145: 1906–1915. 10.1093/brain/awac149. 35472071 PMC9246708

[37] Krieger D, Shepard P, Kontos A, Collins MW, Puccio A, Eagle SR, et al. Sensory driven neurophysiological mechanisms of concussion: a parsimonious and falsifiable theory. Front Neurol 2025; 16: 1547786. 10.3389/fneur.2025.1547786. 40371083 PMC12074929

[38] Kraemer Y, Mäki K, Marinkovic I, Nybo T, Isokuortti H, Huovinen A, et al. Post-traumatic headache after mild traumatic brain injury in a one-year follow up study: risk factors and return to work. J Headache Pain 2022; 23: 27. 10.1186/s10194-022-01398-9. 35183101 PMC8903563

[39] Ashina H, Dodick DW, Barber J, Temkin NR, Chong CD, Adler JS, et al. Prevalence of and risk factors for post-traumatic headache in civilian patients after mild traumatic brain injury: a TRACK-TBI study. Mayo Clin Proc 2023; 98: 1515–1526. 10.1016/j.mayocp.2023.02.026. 37480909

[40] Yue JK, Phelps RR, Hemmerle DD, Upadhyayula PS, -Winkler EA, Deng H, et al. Predictors of six-month inability to return to work in previously employed subjects after mild traumatic brain injury: a TRACK-TBI pilot study. J Concussion 2021; 5: 20597002211007271. 10.1177/20597002211007271. PMC8153496 34046212

[41] de Neeling M, Liessens D, Depreitere B. Relationship between psychosocial and psychiatric risk factors and poor long-term outcome following mild traumatic brain injury: a systematic review. Eur J Neurol 2023; 30: 1540–1550. 10.1111/ene.15713. 36708085

[42] Zahniser E, Nelson LD, Dikmen SS, Machamer JE, Stein MB, Yuh E, et al. The temporal relationship of mental health problems and functional limitations following mTBI: A TRACK-TBI and TED study. J Neurotrauma 2018; 36: 1786–1793. 10.1089/neu.2018.6172. PMC6551992 30543138

